# Magnetoreception in *Cataglyphis hellenica* ants

**DOI:** 10.1007/s00359-026-01794-5

**Published:** 2026-02-05

**Authors:** Chiara Tenneriello, Johanna W. Wegmann, Pauline N. Fleischmann

**Affiliations:** 1 AG Neurosensorik/Animal Navigation, Institute of Biology and Environmental Sciences, Faculty V, Carl von Ossietzky Universität Oldenburg, 26129 Oldenburg, Germany; 2 Behavioral Physiology and Sociobiology (Zoology II), Biocenter, University of Würzburg, 97074 Würzburg, Germany; 3 Research Center Neurosensory Science, Carl von Ossietzky Universität Oldenburg, 26111 Oldenburg, Germany

**Keywords:** Compass orientation, Desert ants, Insect navigation, Learning walks, Magnetic sense, Path integration

## Abstract

Magnetoreception is the ability of animals to detect and use the geomagnetic field (GMF) for spatial orientation. *Cataglyphis* ants are experimental models for insect navigation and magnetoreception. At the beginning of their foraging life, *Cataglyphis* ants perform learning walks (LWs), explorative excursions around the nest, with pirouettes (tight turns about the ants’ body axes). During a pirouette, an ant gazes to the nest entrance, an invisible hole in the ground. Until now, *Cataglyphis nodus* has been the only desert ant species shown to use the GMF to align their gazes to the nest entrance during LW pirouettes. In the present study, we show that *Cataglyphis hellenica*, phylogenetically distant from *C. nodus*, but inhabiting the same environment, also possesses a magnetic sense. When *C. hellenica* ants are exposed to an experimental alteration of the GMF (alteration of the horizontal component about 180° or + 120°), they gaze to the fictive position of the nest entrance. This study demonstrates that *C. hellenica* ants use the GMF to gaze back to the nest entrance, confirming the presence of magnetoreception in a second *Cataglyphis* species, in addition to *C. nodus*. This suggests that the use of the GMF for path integration is rather common than unique in *Cataglyphis* species.

## Introduction

Effective animal navigation is essential for the survival of animals. Animals make use of multimodal cues, including magnetic cues, to find their way. Magnetoreception is the ability of animals to detect the geomagnetic field (GMF) and to use it as a cue for navigation and spatial orientation (Winklhofer 2010). In the last century, the interest in studying how animals use magnetic cues to navigate has increased. The first studies about the use of the GMF discovered that both birds and honeybees possess a magnetic sense (birds (Merkel and Wiltschko 1965), honeybees (Lindauer and Martin 1968)). Since then, many more animal species turned out to be magneto-sensitive, including many invertebrate species, especially diverse insect species (for a review: Vacha 2017).

Desert ants are prime examples of solitary central place foragers and, for that reason, experimental models for insect navigation that have been studied for decades (Wehner 2020). *Cataglyphis* ants rely on path integration during navigation (Müller and Wehner 1988). Path integration is the ability to combine direction and distance information to continuously calculate the shortest way back to the point of origin, the nest (Wehner and Srinivasan 2023). *Cataglyphis nodus* (Brullé, 1833) ants have a magnetic sense: They use artificial magnetic landmarks as nest-defining cues when returning from their food searching (Buehlmann et al. 2012). The GMF is a necessary and sufficient cue to align their gaze directions during learning walks (LWs) (Fleischmann et al. 2018a). LWs are short excursions around the nest entrance, during which the ants do not collect any food, but calibrate their celestial compass system, and learn the landmark panorama (Fleischmann et al. 2017, 2018b). *C. nodus* use GMF in a clear and well-defined behavior: during LWs, ants perform voltes (small, walked circles) and pirouettes (full or partial turns about the ant’s body axis) (Fleischmann et al. 2017). During a pirouette, the ants use the GMF to gaze to the nest entrance, an invisible hole in the ground (Fleischmann et al. 2018a). During a pirouette, the ants stop numerous times and presumably take “snapshots” of the nest surrounding (Zeil and Fleischmann 2019). Until now, *C. nodus* is the only desert ant species that has been shown to be equipped with a magnetic sense and to use the GMF for path integration during LWs (Fleischmann et al. 2018a). Other ant groups perform similar rotational elements, for example, Australian desert ants (Wystrach et al. 2014; Deeti and Cheng 2021; Freas and Cheng 2025), Namibian desert ants (Müller and Wehner 2010) and bull ants (Jayatilaka et al. 2018). In addition, experimental alterations of the magnetic field during LWs affect structural changes in the *C. nodus* ant’s higher-order brain regions, involved in path integration and motor control (central complex), and in sensory integration and memory formation (mushroom bodies) (Grob et al. 2024a). Furthermore, *C. nodus* ants possess a polarity-sensitive magnetic compass, i.e., they utilize the polarity of the magnetic field to orient (Grob et al. 2024b). Whether other *Cataglyphis* ants possess a magnetic sense has been unknown, leaving the question open whether magnetoreception is a unique characteristic or a more general feature in the *Cataglyphis* genus.

**Fig. 1:**
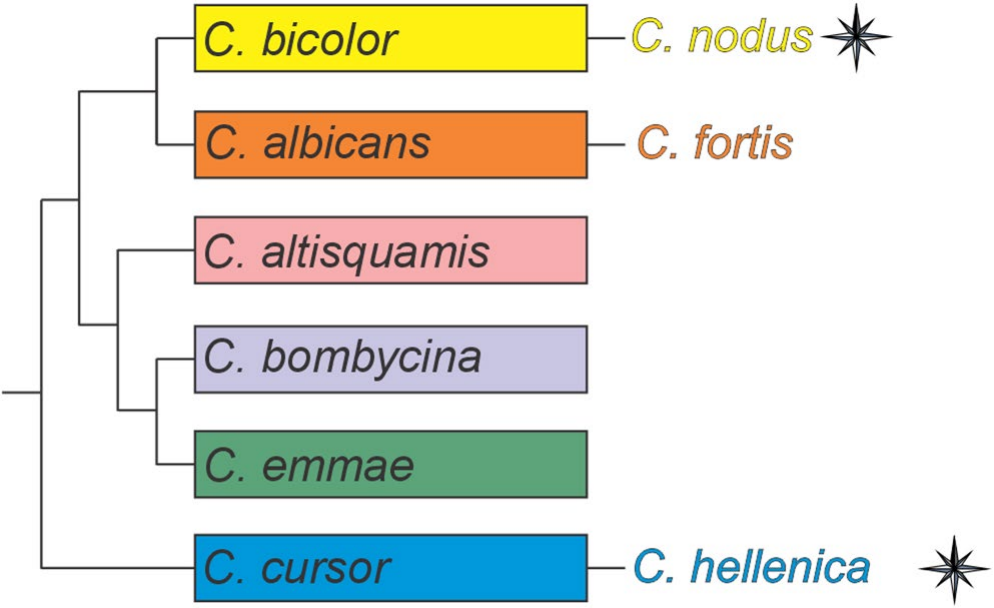
Phylogenetic relationship between *Cataglyphis* species groups, with focus on *C. nodus (C. bicolor* group*)*, *C. fortis (C. albicans* group*)* and *C. hellenica (C. cursor* group). *C. nodus* and *C. hellenica* have been shown to have a magnetic sense (indicated by the compass rose icon). Phylogeny based on Wehner 2020, p. 41, Fig. 2.12

In the cluttered pine forest in Greece, there live two *Cataglyphis* species: *C. nodus* and *Cataglyphis hellenica* (Forel, 1886). *C. hellenica* ants perform LWs with voltes and pirouettes (Fleischmann et al. 2017). As *C. nodus*, *C. hellenica* ants stop numerous times during pirouettes, and during the longest stopping phase, they gaze to the nest entrance, an invisible hole in the ground (referred to as *Cataglyphis aenescens,* Fleischmann et al. 2017). From a phylogenetic perspective, they are distant (Fig. 1). *C. nodus* is part of the *C. bicolor* group, whereas *C. hellenica* is part of the *C. cursor* group. Ant workers of the *C. bicolor* group show a higher brain-body size allometry, compared to *C. aenescens* workers that also belong into the C. cursor group, which is probably related to the sizes of the colonies (Wehner et al. 2007). Colonies of *C. aenescens* contain only a few hundred individuals, while in *C. bicolor*, the number of workers range in the thousands (Wehner et al. 2007).

In the present study, we investigate the effect of different experimental magnetic conditions on the LW behavior in *C. hellenica* ants. The horizontal component of the GMF was turned about 180° and + 120° to test whether ants, during LW pirouettes, turn their gaze directions towards the respective fictive position of the nest entrance. We show that *C. hellenica* ants have a magnetic sense, and they use the GMF to align their gazes back to the nest entrance.

## Materials and methods

### Study animals and the field location

The experiments took place in two field seasons in June and July 2024 and 2025, in Marathon, Greece. We used two different nests of *C. hellenica* ants (Forel, 1886, Fig. 2a) in their natural environment. This species was referred to as *C. aenescens* before (Borowiec and Salata 2021). *C. hellenica* and *C. aenescens *were synonymized in 2013 (Borowiec and Salata 2013), but a genetic study revealed that *C. aenescens* actually contains several cryptic species that are distinct from the true *C. aenescens *described from Southern Russia (Kuhn et al. 2020). For that reason, the species has been redescribed as *C. hellenica* (Borowiec and Salata 2022).At the beginning of both experiments, we use the first three days to mark all the ants coming out of the nest entrance with iron oxide-free nail polish (2024: violet (no. 152); 2025: white (no. 134), gitti GmbH, Berlin, Germany). Marked ants were considered experienced and, for that reason, not included in the experiments. Only unmarked naïve ants coming out of the nest entrance were included in the experiments.

**Fig. 2:**
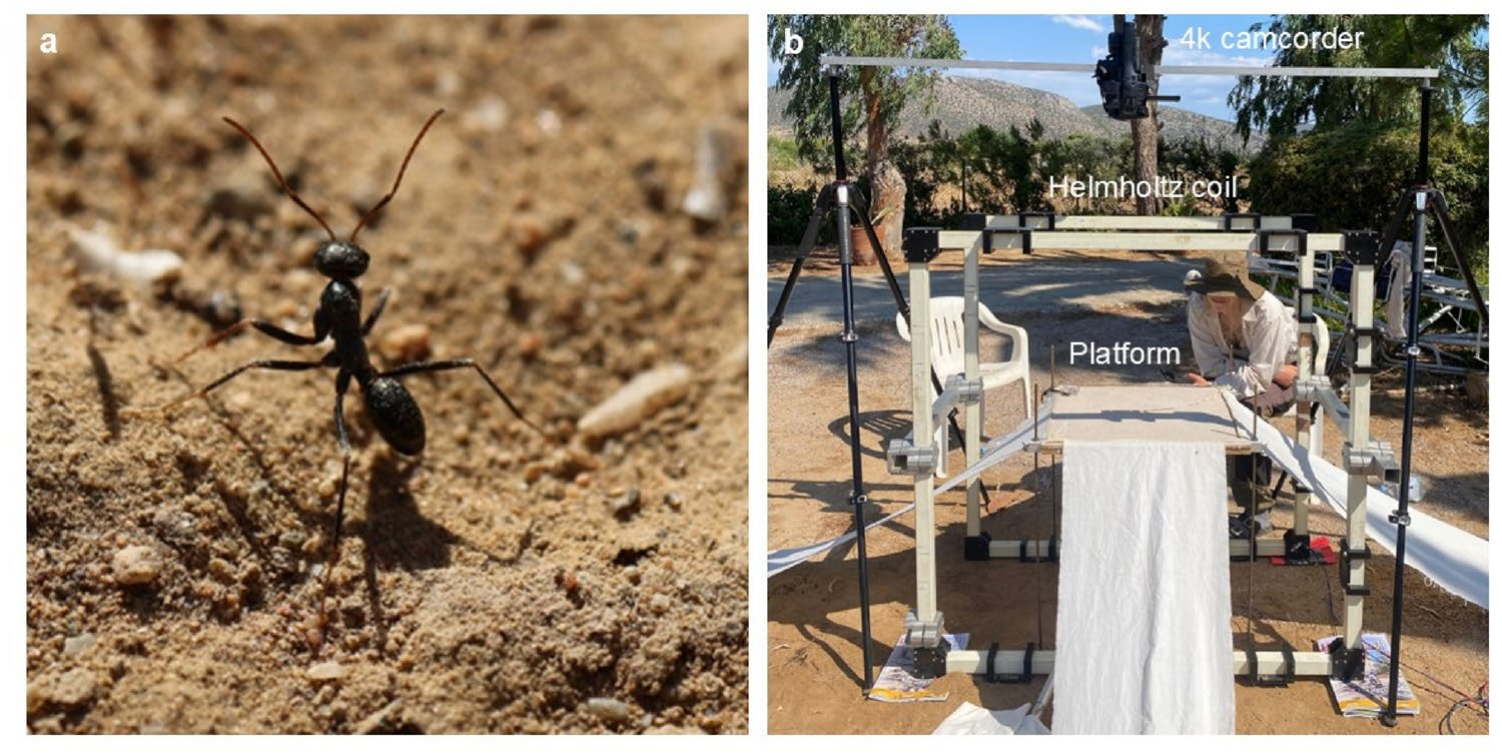
Experimental animal and experimental setup. **a** *C. hellenica* worker next to the nest entrance. b Helmholtz coil setup with a Helmholtz coil surrounding the experimental platform and a 4k camera held by two tripods. The ants’ nest was connected to the center of the platform via tunnels and tubes

### Experimental setup

To test whether *C. hellenica* ants possess a magnetic sense and whether they use the GMF as a compass cue for orientation during their LWs. *C. hellenica* ants performed LWs in the Helmholtz coil setup (Fleischmann et al. 2018a). In the first experiment, performed in 2024, ants were exposed to an alteration of the horizontal component of the magnetic field about 180°. In the second experiment, performed in 2025, ants were exposed to a change of + 120°. The experimental setup (Fig. 2b) consists of a rectangular Helmholtz coil (HHS 5213-50, Middle coil, Schwarzbeck Mess-Elektronik, Schönau, Germany), connected to a customized DC power supply, and a platform with four ramps connecting the platform to the ground (for additional technical information about the Helmholtz coil, see the coil sheet in the supplementary material). The platform was placed at the center of the coil system, because the homogeneity of the magnetic field is highest in the center of the coil system. The Helmholtz coil setup was leveled and north oriented. The natural nest entrance was covered with a cylinder with a lid (nest cover) connected via a flexible tube to the center of the platform. In this way, the ants were leaving the nest entrance through the nest cover to the platform (60 cm × 60 cm). Experienced foragers left the platform using the fabric ramps.

We used a magnetometer (MEDA FVM400 vector magnetometer, Inc. Macintyre Electronic Design Associates, Inc. 43676 Trade Center Place, Suite 145, Dulles, VA 20166) to measure the GMF and the experimentally altered magnetic field. The measurements are summarized in Table 1.

**Table 1:** One-time measurements as displayed by the magnetometer of magnetic fields on the experimental platform in the center of the coil before the alteration (GMF) and after the alteration (experimental magnetic field condition)

		Declination, D (°)	Inclination I (°)	Total intensity B (µT)	X component (µT)	Y component (µT)	Z component (µT)
Experiment 1	Before 180° alteration	0.0	56.0	48.095	26.824	− 0.022	39.922
	After 180° alteration	179.6	55.5	48.193	27.241	0.161	39.767
Experiment 2	Before + 120° alteration	0.0	55.0	46.323	26.551	0.015	37.955
	After + 120° alteration	118.1	53.0	46.957	− 13.130	25.035	37.505

### Magnetic manipulations

**Fig. 3:**
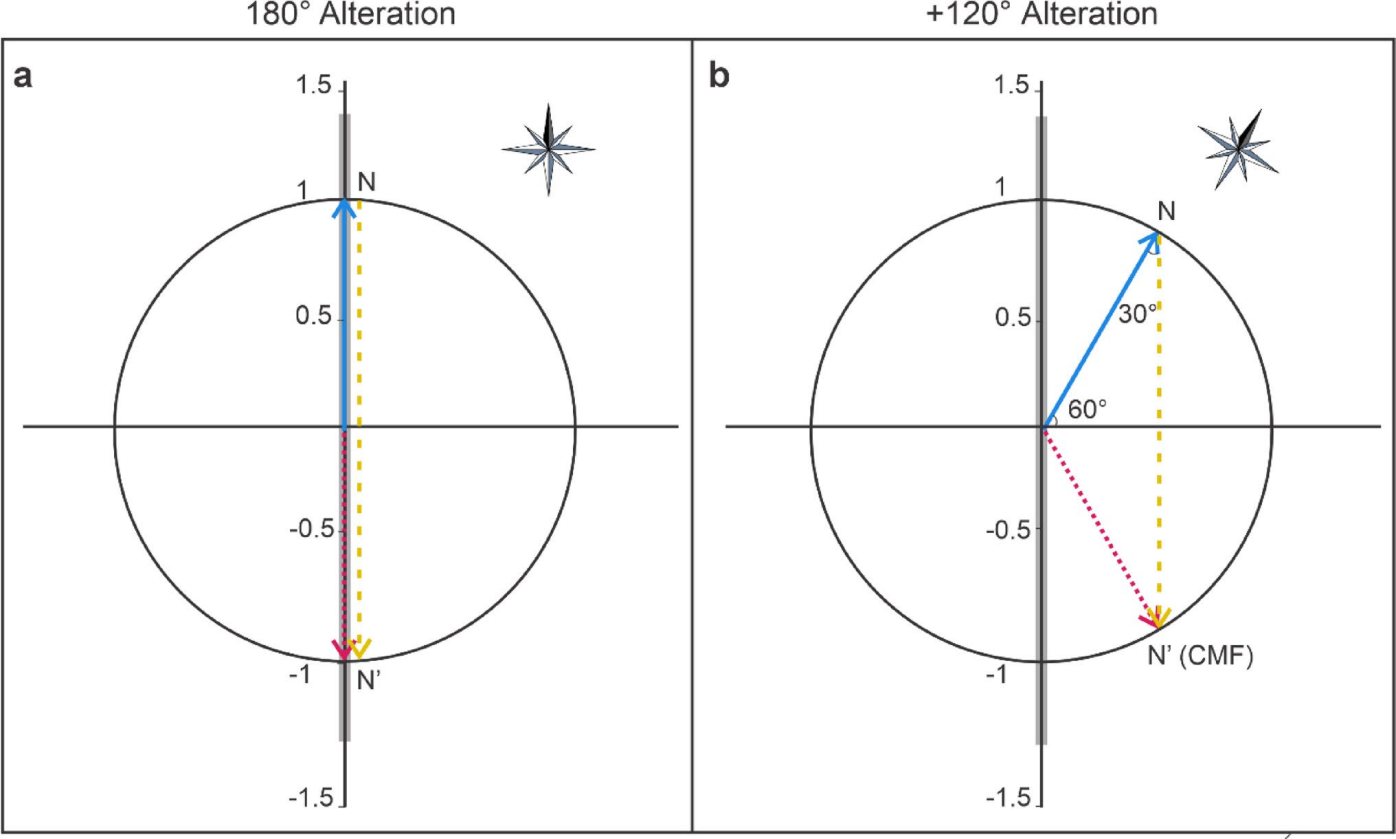
Experimental manipulations of the magnetic field conditions: **a** 180° alteration and **b** + 120° alteration. The coil axis (grey bold line) has different orientations relative to the GMF in a and b. The horizontal component of the GMF (blue straight line) points towards geomagnetic north (N), with a strength of B_H−GMF_. The horizontal component of the experimentally altered magnetic field (red dotted line) points towards the changed magnetic north (N’ (CMF)), with a strength (B_CMF_). The horizontal component of the experimentally altered magnetic field is given by the superposition, i.e., given by a vector addition, of the horizontal component of the GMF and the magnetic field generated by the Helmholtz coil (yellow dashed line). The compass rose is aligned with geomagnetic North

In our experiment, we exposed *C. hellenica* to the manipulated magnetic field conditions most commonly used in the magnetoreception literature: a total magnetic field, i.e., a superposition of the GMF and an artificially generated magnetic field, whose horizontal magnetic field component is altered with respect to the horizontal component of the GMF either about 180° (e.g. spiny lobsters (Lohmann et al. 1995); bats (Wang et al. 2007); desert ants (Fleischmann et al. 2018a, 2022; Grob et al. 2024a; 2024b)); or about + 120° (e.g. night migratory songbirds (Wiltschko and Wiltschko 1988; Leberecht et al. 2022); Australian Bogong Moth (Dreyer et al. 2018); Migratory bats (Schneider et al. 2023)). These manipulated magnetic field conditions will, in the following, be referred to as 180°/+120° alteration, respectively. To realize the 180° alteration (Fig. 3a), we aligned the Helmholtz coil axis (the axis through the center of both coils of a Helmholtz coil pair, i.e. an axis parallel to the magnetic field generated by the coil) north, i.e., such that the artificial magnetic field generated in the center of the Helmholtz coil aligns with the north-south direction. Current was then passed through the Helmholtz coil, generating an artificial magnetic field B_coil_ whose strength is twice the strength of the horizontal component of the GMF (B_H−GMF_). This may be expressed by the following formula:

$$\:{\mathrm{B}}_{\mathrm{c}\mathrm{o}\mathrm{i}\mathrm{l}}=2\times\:{\mathrm{B}}_{\mathrm{H}-\mathrm{G}\mathrm{M}\mathrm{F}}$$

To realize the + 120° alteration (Fig. 3b), we rotated the Helmholtz coil in the horizontal plane about 60° from the north-south direction and passed a current through the Helmholtz coil suitable to generate an artificial magnetic field B_coil_ whose strength is given by the following formula:

$$\:{\mathrm{B}}_{\mathrm{c}\mathrm{o}\mathrm{i}\mathrm{l}}\:=\:2\:\times\:\mathrm{cos}\left(30^\circ\!\!\:\right)\times\:\:{\mathrm{B}}_{\mathrm{H}-\mathrm{G}\mathrm{M}\mathrm{F}}$$

This condition for the artificial magnetic field follows directly from Fig. 3b, which illustrates the magnetic field vectors of the GMF (blue), the magnetic field generated by the Helmholtz coil (yellow), and the total magnetic field (red) resulting from the alteration. These three vectors may be considered as forming an isosceles triangle, which can further be subdivided into two right triangles. In a right triangle, the cosine of an acute angle α (here: α = 30°) is equal to the ratio between the length of the adjacent side (here: 1/2 B_coil_) to that angle and the length of the hypotenuse (here: B_H−GMF_), i.e. $\:\mathrm{c}\mathrm{o}\mathrm{s}\left(30^\circ\:\right)=\frac{1}{2}\:{\mathrm{B}}_{\mathrm{c}\mathrm{o}\mathrm{i}\mathrm{l}}\:/\:{\mathrm{B}}_{\mathrm{H}-\mathrm{G}\mathrm{M}\mathrm{F}}$.

Using a + 120° alteration of the horizontal component of the magnetic field differed from our previous experiments (Fleischmann et al. 2018a, 2022; Grob et al. 2024b). In contrast to a 180° alteration, the + 120° alteration has the advantage that it can be realized using a conventional compass and, notably, without using a magnetometer or moving the Helmholtz coil. It is to align the coil to 60° from the north-south direction and to increase the current of the coil until the compass shows a change of + 120° with respect to the natural magnetic field conditions. The alignment of the coil will ensure that the strength of the experimental magnetic field will be correct, i.e., will be equal to the local geomagnetic field strength (See Fig. 3b). This is not possible with the 180° alteration, because a current of any value passed through the Helmholtz coil will lead to a change in the total magnetic field directly alongside the North-South axis. Therefore, a compass would either point directly north or directly south, switching approximately when the horizontal component of the magnetic field is eliminated by the coil. In addition, the + 120° alteration can be useful because some animals make mistakes in the opposite direction (bimodal distributions). For example, desert ants (*C. fortis*) show a bimodal distribution of their homing directions under certain polarization patterns (Lebhardt et al. 2012). To avoid confusion, it is preferable to select a value (e.g., 120°) far enough from the natural direction to prevent overlapping confidence intervals, but still not opposite to it.

### Video recordings

To record the LWs of naïve ants, we installed a 4 K camcorder (HCX1000, Panasonic Corporation, Kadoma, Japan) above the platform and the Helmholtz coil (Fig. 2b). The camera was connected to a Cat S60 smartphone (Caterpillar, Peoria, USA) with the Panasonic Image App (Version 10.9.2, Panasonic Corporation, Kadoma, Japan). An observer sitting close to the setup started a video recording (50 fps) every time an unmarked ant was leaving the nest entrance. After a naïve ant performed at least one pirouette, the Helmholtz coil was turned on, and the horizontal component of the magnetic field was experimentally altered (180° or + 120° alteration). For each experimental animal, one pirouette was also recorded under experimental magnetic conditions. The video recording was stopped when the naïve ant returned to the nest entrance or left the platform.

### Data analysis

We used the application Free Video to JPG Converter (v. 5.0.101 build 201, DVDVideoSoft, DIGITAL WAVE LTD., London, UK) to convert the videos into JPEG image stacks. The videos were manually analyzed frame by frame with the application DIGILITE (Jan Hemmi & Robert Parker, Australian National University) in MATLAB R2024a (MathWorks, Natick, MA, United States). We marked the positions of the mandibles and of the thorax during a pirouette of each ant in each frame. In addition, we marked the position of the nest entrance for every pirouette. For every ant used in the analysis, we analyzed one pirouette before (defined as the first LW pirouette after the coil was switched on at least 5 cm distant from the nest entrance) and the subsequent pirouette after the magnetic alteration. After that, a customized script in MATLAB calculated the fictive nest entrance position for each experimental ant (Fleischmann et al. 2018a). The fictive nest entrance was calculated by rotating the mandibles-nest vector by the degree of the magnetic alteration (experiment 1: 180 °, experiment 2: +120°) when the coil was switched on. The script then calculated the gaze directions during LW pirouettes relative to the nest entrance for pirouettes before the Helmholtz coil was switched on or relative to the nest entrance and relative to the fictive position of the nest entrance (for pirouettes after the Helmholtz coil was switched on) (Fleischmann et al. 2018a, 2022; Grob et al. 2024a; b). The relative gaze direction during the longest stopping phase (a stop of forward and rotational movement with a constant gaze direction (+/-10° for at least 100 ms)) of a pirouette was determined by our MATLAB script and used for statistical analyses (Fleischmann et al. 2017).

We cannot state that the analysis is blind, because the analyzer knows (and must know) which kind of experiment is analyzed. However, while tracking the pirouettes, the analyzer cannot see the position of the points of interest (position of the nest entrance and of the fictive nest entrance, respectively). For this reason, we are confident that there is no bias towards the position of the nest or fictive nest entrance. The position of the fictive nest entrance is only calculated after the analysis is done.

### Statistical analysis

We used the software Oriana 4.02 (Kovach Computing Services, Anglesey, UK) for the circular statistics analysis. We performed the Rayleigh Uniformity test (significance level: 0.05) to analyze whether the gaze directions were randomly distributed or significantly directed. The gaze directions were grouped into 10°-bins for plotting. When the gaze directions were significantly directed, we calculated the 95% confidence interval (95% CI) to check whether the position of interest (nest entrance or fictive nest entrance defined as 180°) was included within the interval limits. We did not include the 95% CI when it was not reliable due to low concentration. We used pairwise Mardia–Watson–Wheeler test (significance level: 0.05) to compare distributions of the gaze directions before and after the manipulation relative to the nest entrance.

## Results

### Experiment 1: change of 180 degrees of the horizontal component of the GMF

**Fig. 4:**
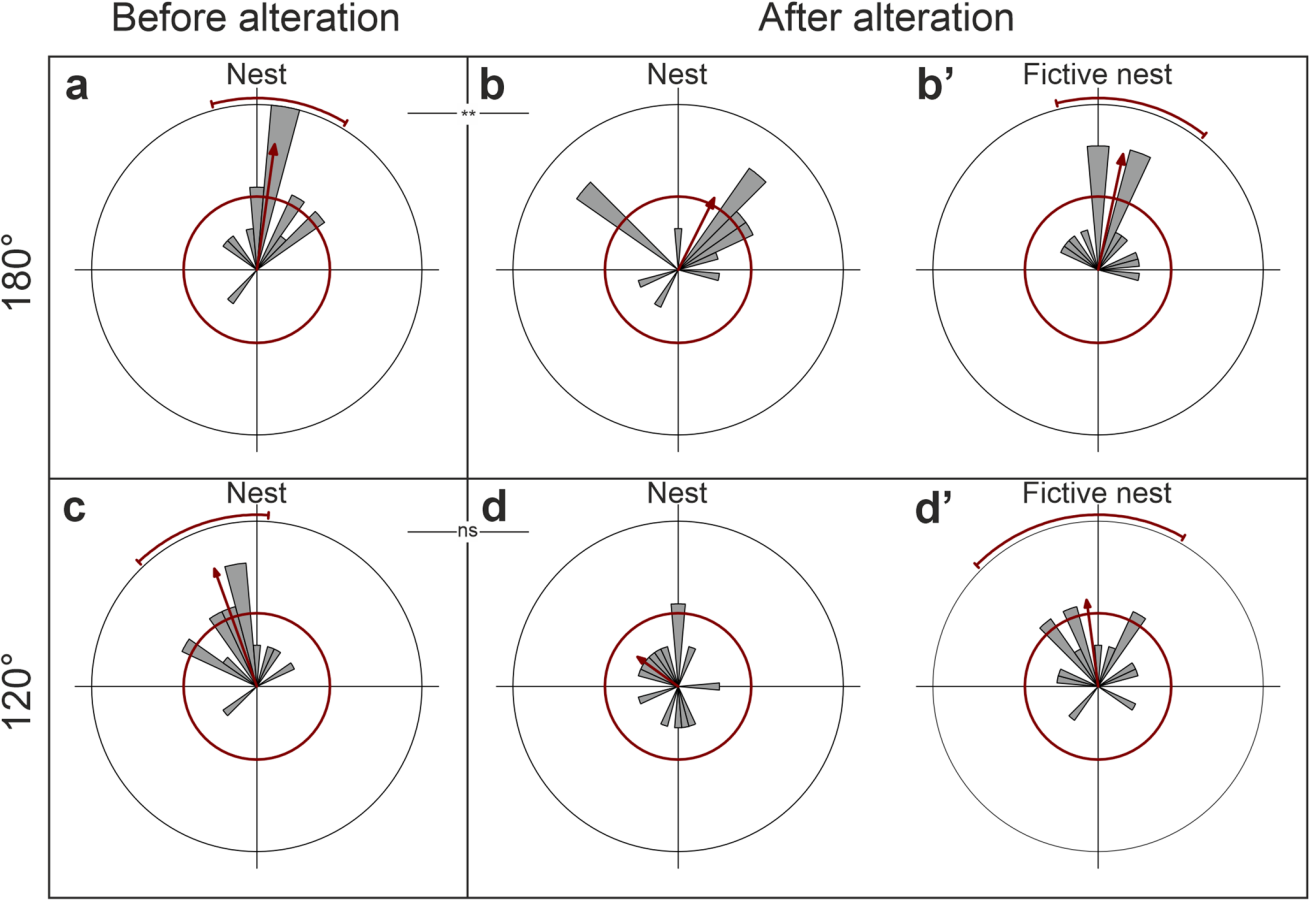
Gaze directions of naïve ants (*C. hellenica*) before and after the experimental alteration of the horizontal component of the GMF. **a** and **b** Gaze directions of naïve ants before and after a 180° alteration of the GMF (**a** *n* = 15, **b** *n* = 15). **c** and **d** Gaze directions of naïve ants during before and after a + 120° alteration of the GMF (**c** *n* = 15, **d** *n* = 15). **b′ ** and ** d′** Same data as in **b**,** d**) relative to the fictive nest entrance. The data of the gaze directions of the ants are shown in grey, with bins including 10°. The corresponding statistics of the Rayleigh uniformity test and the confidence interval are indicated in red: the circle represents the significance level (α = 0.05), the arrow represents the r-vector pointing to the mean gaze direction, and the arc represents the 95% confidence interval. When the arrow exceeds the circle, the data is significantly directed. When the goal direction (in this case: nest or fictive nest) is included in the confidence interval, the gaze direction is directed towards the goal direction. The outer circle indicates tic 4

We exposed *C. hellenica* ants to a 180° alteration of the horizontal component of the magnetic field, to test whether the ants use the GMF to gaze back to the nest entrance. During the pirouette before the Helmholtz coil was switched on, i.e., under natural GMF conditions, *C. hellenica* ants were gazing to the nest entrance in the experimental platform (Fig. 4a, Rayleigh test Z = 8.845, n = 15, p < 0.001, r = 0.768, mean vector (µ) = 188°, CI 95% = 165° − 211°). When the horizontal component of the magnetic field was changed by 180°, the ants’ gaze directions were directed towards the fictive nest entrance (Fig. 4b’, Rayleigh test Z = 7.772, *n* = 15, *p* < 0.001, *r* = 0.717, mean vector (µ) = 192°, CI 95% = 166° – 218°). Gaze directions were slightly directed when plotted towards the nest entrance (Fig. 4b, Rayleigh test Z = 3.554 *n* = 15, *p* = 0.026, *r* = 0.487, mean vector (µ) = 207°). The gaze directions relative to the nest entrance were significantly different between the pirouettes before and after the manipulation (Fig. 4a-b, N_Pb_nest_ = 15, N_Pa_nest_ = 15, W = 10.637, *p* = 0.005).

### Experiment 2: change of + 120 degrees of the horizontal component of the GMF

In the second experiment, we exposed the experimental animals to a + 120° alteration of the horizontal component. Ants gazed to the nest entrance under GMF condition (Fig. 4c, Rayleigh test Z = 8.625, n = 15, p < 0.001, r = 0.758, mean vector (µ) = 160°, CI 95% = 136° – 184°). When the horizontal component of the GMF was altered with a change about + 120°, the ants’ gaze directions were not directed towards the nest entrance in the experimental platform (Fig. 4d, Rayleigh test Z = 1.395 n = 15, p = 0.251, r = 0.305, mean vector (µ) = 126°), but towards the fictive nest entrance (Fig. 4d’, Rayleigh test Z = 4.198, *n* = 15, *p* = 0.013, *r* = 0.529, mean vector (µ) = 172°, CI 95% = 135° – 210°). The gaze directions relative to the nest entrance were not significantly different in the pirouettes before and after the change of the magnetic field (Fig. 4c-d, N_Pb_nest_ = 15, N_Pa_nest_ = 15, W = 4.091, *p* = 0.129).

## Discussion

*C. hellenica* ants possess a magnetic sense, and they use the GMF to gaze back to the nest entrance during LW pirouettes. Our study shows that magnetoreception is present in a second species of *Cataglyphis* ants, providing the first evidence that the use of geomagnetic cues for navigation is more common than exceptional in the *Cataglyphis* genus. *C. hellenica* ants gaze to the nest entrance during natural GMF conditions and to the fictive position of the nest entrance when the horizontal component of the GMF has been experimentally altered, as do *C. nodus* ants (Fleischmann et al. 2018a).

*Cataglyphis* ants align their gaze directions towards the fictive position of the nest entrance regardless of the experimentally induced magnetic field alteration (180°: Fleischmann et al. 2018, Grob et al. 2024b; +90° and − 90°: Fleischmann et al. 2018, + 120°: present study). It is noteworthy here that the spatial relationship between the gaze directions towards the position of the nest entrance and towards the fictive position of the nest entrance is almost never the same as the experimentally induced change, e.g., 180° in the 180° alteration experiment, because it depends on the current position of the ant where it performs a pirouette. For that reason, a bimodal distribution of data, e.g., with gazes to nest and anti-nest direction, will not be confused with ants gazing to the fictive position of the nest entrance. The experimentally induced alterations, i.e., the rotation of the horizontal component of the experimental magnetic field with respect to the natural GMF, in future experiments can thus be a free choice. From a practical point of view, portable magnetometers are available nowadays and make it unnecessary to move the Helmholtz coil to determine the correct settings. Furthermore, the 180° alteration has practical advantages, it is intuitive and unambiguous. This is especially helpful for experimental setups that have to be built and removed every day. The 180° alteration thus is ideal for experiments with *Cataglyphis* to test the role of the magnetic sense for path integration during LW pirouettes.

*C. hellenica* and *C. nodus* ants both use the GMF to align their gaze directions towards the nest entrance during LW pirouettes (Fleischmann et al. 2018a). This is an important result to understand how magnetoreception is spread and how it evolved in the *Cataglyphis* genus, because these two species belong to two phylogenetically distant groups in the genus (Knaden et al. 2012). *C. hellenica* and *C. nodus* live in the same environment, the Greek cluttered pine forest (Wehner 1987; Borowiec and Salata ). These findings open up questions about the use of magnetoreception in different *Cataglyphis* species and its evolutionary distribution across the genus. *Cataglyphis* ants are scavengers that forage at the hottest time of the day. They collect dead arthropods during their long foraging trips (Wehner 1987). Differently from *C. nodus* and *C. hellenica*, the desert ant *Cataglyphis fortis* (Forel, 1902), which lives in the salt pans of Algeria and Tunisia, an environment without any prominent visual landmarks, performs LWs with only voltes and no pirouettes (Fleischmann et al. 2017). This is likely due to the homogeneity of its natural habitat, where the absence of distinctive landmarks reduces the efficiency of capturing snapshots of the panorama (Fleischmann et al. 2017). While *C. nodus* and *C. hellenica* are distantly related species that rely on the GMF during the gaze back to the nest behavior, *C. nodus* and *C. fortis* are more closely related. *C. fortis* is part of the group *C. albicans* (for phylogenetic relation between the species see Fig. 1) (Knaden et al. 2012). However, *C. fortis* ants do not display the gaze back behavior during LWs, although this does not preclude their use of the GMF. The demonstration of magnetoreception in both *C. nodus* and *C. hellenica* ants, i.e. two groups that are phylogenetically distant in the genus, suggests that magnetoreception is an ancestral trait, potentially serving broader navigational functions. This may include calibration of the time-compensated celestial compass system, straight-line orientation in darkness, or other navigational tasks (Fleischmann et al. 2020). The phylogenetic relations between the *Cataglyphis* species studied suggest that *C. fortis* may also use the GMF for navigation, even though they do not perform any pirouettes in their LWs.

Until now, *C. nodus* and *C. hellenica* are the only ants that are shown to use the GMF for path integration during LWs under natural conditions, i.e. ants of both species use the GMF to gaze to the nest entrance while performing LW pirouettes. There is evidence that other ant species are magneto-sensitive. Leaf-cutter ants (*Atta colombica*), respond to magnetic reversal under an overcast sky (Banks and Srygley 2003; Riveros et al. 2014). The authors suggest that leaf-cutter ants use the GMF as a reference for path integration to go back home (Riveros and Srygley 2008). Also, two species of wood ants (*Formica rufa* and *Formica pratensis*) rely on the GMF when re-visiting a feeder and all other navigational cues are not available (Camlitepe and Stradling 1995; Camlitepe et al. 2005). *F. rufa* can also learn a direction based on magnetic information during foraging trips under laboratory conditions (Collett and Philippides 2022). In addition, Pereira et al. (2019) showed that two species of solitary foraging ants (*Ectatomma brunneum* and *Neoponera inversa*) reduce their foraging abilities when exposed to altered magnetic fields. Not only ants, but also other Hymenoptera rely on the GMF (Fleischmann et al. 2020). For example, honeybees (*Apis mellifera*) are magneto-sensitive. In their waggle dance on the vertical comb, there is a systematic deviation changing over time (the residual misdirection) that was found to depend on the GMF (Lindauer and Martin 1968). However, until now, the LW pirouettes in *C. hellenica* and *C. nodus* are the only behavior where GMF is used as a necessary and sufficient cue under natural conditions when all other cues are available for navigation.

Many ant species perform LWs at the beginning of their foraging life and live in a cluttered forest-like environment (Zeil and Fleischmann 2019). Australian desert ants (*Melophorus bagoti*) use three to four days at the beginning of their foraging life to perform LWs, during which they stop different times and perform pirouettes (Deeti and Cheng 2021). In addition, *M. bagoti* foragers in their outbound trips perform turns to gaze back to the nest entrance (Freas and Cheng 2025). *M. bagoti* ants also perform scanning behaviours with pauses separated by fast rotations displayed at different situations, for example, at their first departures from their nest, each morning before foraging or when the panorama has been changed experimentally (Wystrach et al. 2014). In *C. nodus*, initial LWs and relearning walks (reLWs) share similarities, but also have unique characteristics (Fleischmann et al. 2022). For example, both include pirouettes, but each initial LW ends in the nest, whereas typically a reLW continues as a foraging trip. Namibian desert ants, *Ocymyrmex robustior*, perform reLWs with rotations like pirouettes during which ants stop different times. During the stopping phases, the ants orient towards the nest entrance (Müller and Wehner 2010; Wystrach et al. 2014). Both species live in a cluttered environment, a semi-arid desert habitat dominated by grass, bushes, and trees (Marsh 1985; Muser et al. 2005). Wood ants (*Formica rufa*) perform reLWs while returning to their nest after discovering a new food source, during which they turn back and look to landmarks close to the feeder (Nicholson et al. 1999). Importantly, *C. nodus* ants only use the GMF as a reference system during initial LWs and discard magnetic information during reLWs (Fleischmann et al. 2022). For this reason, it will be particularly interesting to check the use of the GMF by the ant species that perform LWs including looks back to the nest at the beginning of their foraging careers. Bull ants perform LWs (e.g., *Myrmecia croslandi* (Jayatilaka et al. 2018) with similar features as *C. nodus* (Zeil and Fleischmann 2019). They also walk around the nest entrance and perform rotational elements when becoming foragers.

## Data Availability

Data is available online: 10.57782/8LWWEQ

## Abbreviations

LWs: Learning walks
reLWs: Re-learning walks
GMF: Geomagnetic field
